# Tobacco Smoking and Lung Cancer Risk After Negative Baseline Low-Dose Computed Tomography Findings

**DOI:** 10.1001/jamanetworkopen.2026.1913

**Published:** 2026-03-20

**Authors:** Yin Liu, Xiaoli Guo, Ranran Qie, Qiong Chen, Huifang Xu, Xiaoyang Wang, Hongwei Liu, Hong Wang, Ruihua Kang, Mengfei Zhao, Cheng Cheng, Liyang Zheng, Shuzheng Liu, Jinyu Zhang, Xinying Yue, Youlin Qiao, Shaokai Zhang

**Affiliations:** 1 Department of Cancer Epidemiology, The Affiliated Cancer Hospital of Zhengzhou University & Henan Cancer Hospital, Zhengzhou, China; 2 Center for Global Health, School of Population Medicine and Public Health, Chinese Academy of Medical Sciences and Peking Union Medical College, Beijing, China

## Abstract

**Importance:**

Lung cancer (LC) remains the leading cause of cancer-related mortality worldwide, with tobacco smoking being the primary risk factor. However, the long-term LC risk among individuals with negative low-dose computed tomography (LDCT) findings and the role of tobacco smoking in risk stratification remain poorly understood, limiting evidence-based guidance for subsequent screening intervals.

**Objective:**

To evaluate the association of tobacco smoking with long-term LC risk after a negative baseline LDCT finding and to inform optimized screening strategies.

**Design, Setting, and Participants:**

This population-based, prospective cohort study was conducted under the Cancer Screening Program in Urban China. Individuals aged 40 to 74 years with negative baseline LDCT findings (October 1, 2013, to December 31, 2021) were included, with follow-up until December 2023. All participants were monitored for LC incidence.

**Exposures:**

Self-reported smoking status, pack-years, and time since quitting.

**Main Outcomes and Measures:**

The primary outcome was LC incidence, analyzed using Kaplan-Meier methods and multivariable Cox proportional hazards regression models. The association between smoking exposure and LC risk was assessed, with time-stratified analyses and dose-response associations.

**Results:**

Among 30 565 participants (14 761 never smokers and 15 804 smokers; mean [SD] age, 57.1 [7.7] years; 15 693 [51.3%] female), 76 LC cases occurred during 139 011.51 person-years (crude incidence rate, 54.67 of 100 000 person-years). Smokers had higher LC risk than never smokers (adjusted hazard ratio [AHR], 2.73; 95% CI, 1.49-5.01), driven by those with a smoking history of 20 pack-years or more (eg, ≥30 pack-years: AHR, 3.22; 95% CI, 1.85-5.58). There was no elevated risk at 2 years (AHR, 2.07; 95% CI, 0.91-4.69), but risk was significantly increased at 3 years (AHR, 2.54; 95% CI, 1.19-5.41) and onward. A nonlinear dose-response association was found between pack-years and LC risk, with risk surpassing clinically relevant thresholds at approximately 20 pack-years (eg, 20 to <30 pack-years: AHR, 2.48; 95% CI, 1.14-5.40). Females exhibited higher susceptibility than males at comparable exposure (≥30 pack-years: AHR, 5.78 [95% CI,1.87-17.83] for females vs 1.36 [95% CI, 0.18-10.39] for males). Significant risk was seen in those aged 50 to 54 years (≥30 pack-years) and 55 to 74 years (≥20 pack-years). Short-term cessation (<15 years) was not significantly associated with reduced LC risk.

**Conclusions and Relevance:**

In this cohort study, smokers with negative baseline LDCT findings exhibited a substantially elevated long-term LC risk, which became significant only after 2 years after screening. These findings suggest support for extending the initial screening interval and implementing personalized long-term monitoring based on smoking history.

## Key Points

**Question:**

What is the association of tobacco smoking status, pack-years, and cessation duration with long-term lung cancer risk after negative baseline low-dose computed tomography (LDCT) findings?

**Findings:**

In this cohort study of 30 565 participants with negative baseline LDCT findings, smokers, especially those with a smoking history of 20 pack-years or more, had a significantly higher lung cancer risk than never smokers by year 3 after initial screening, with higher susceptibility in women and no significant risk reduction with short-term cessation (<15 years).

**Meaning:**

These findings suggest support for extending the initial screening interval and implementing personalized long-term monitoring based on smoking history.

## Teaser

This cohort study examines the association between smoking exposure and lung cancer incidence among individuals with negative findings on baseline low-dose computed tomography screening.

## Introduction

Lung cancer (LC) is the leading cause of cancer-related mortality globally, with an estimated 1.8 million deaths annually.^1^ In China, an estimated 733 291 LC deaths occurred in 2022, accounting for 40.3% of the global total LC deaths.^2^ Low-dose computed tomography (LDCT) screening has been determined to improve early detection and reduce mortality among high-risk populations.^3,4^ However, approximately 80% of baseline screens yield negative results, even among individuals at high risk of LC,^3,5,6^ leaving a large cohort whose long-term LC risk trajectories remain unclear.

Tobacco smoking remains the predominant cause of LC, contributing to 80% to 90% of cases globally.^7^ Although the dose-dependent relationship between smoking exposure and LC risk has been well established,^8,9^ how smoking exposure is associated with subsequent LC risk after negative LDCT findings is poorly understood. Although the National Lung Screening Trial reported lower LC incidence among heavy smokers with a negative baseline screen result,^10^ it failed to quantify how smoking shapes the LC risk over time. This gap hinders evidence-based tailoring of surveillance intervals.

Clinical guidelines further reflect this uncertainty, exhibiting substantial variability in LDCT screening recommendations.^11^ For example, the United States Preventive Services Task Force (USPSTF) 2021 guideline recommends screening individuals aged 50 to 80 years with a smoking history of 20 pack-years or more who currently smoke or quit within 15 years.^12^ In China, the 2021 guideline targeted those aged 50 to 74 years with a smoking history of 30 pack-years or more,^13^ whereas the 2024 updated lowered the threshold to 20 pack-years or more,^14^ highlighting ongoing debate over optimal eligibility. Furthermore, although annual screening is commonly recommended, evidence suggests extending the interval may enhance cost-effectiveness without compromising outcomes.^15,16,17^ Integrating smoking cessation into screening programs also improves cost-effectiveness,^18,19^ yet the necessary cessation duration for meaningful risk reduction remains unclear. Therefore, population-based studies are urgently needed to inform guideline refinement.

To address this knowledge gap, we examined the association between smoking exposure (status, pack-years, and cessation) and LC incidence among individuals with negative LDCT findings and characterize the temporal evolution of LC risk after negative screening findings. This study aimed to generate actionable evidence to refine LDCT screening guidelines and guide targeted tobacco control strategies for this understudied population.

## Methods

### Study Design and Participants

This population-based, prospective cohort study was conducted under the Cancer Screening Program in Urban China (CanSPUC). The program targets residents aged 40 to 74 years, with a focus on the 5 most prevalent cancers (LC, female breast cancer, liver cancer, upper gastrointestinal tract cancer, and colorectal cancer) in urban China. Detailed descriptions of CanSPUC have been published previously.^20^ The study was approved by the ethics committee of Henan Cancer Hospital. Written informed consent was obtained from all participants. The study followed the Strengthening the Reporting of Observational Studies in Epidemiology (STROBE) reporting guideline.^21^

**Figure 1.**
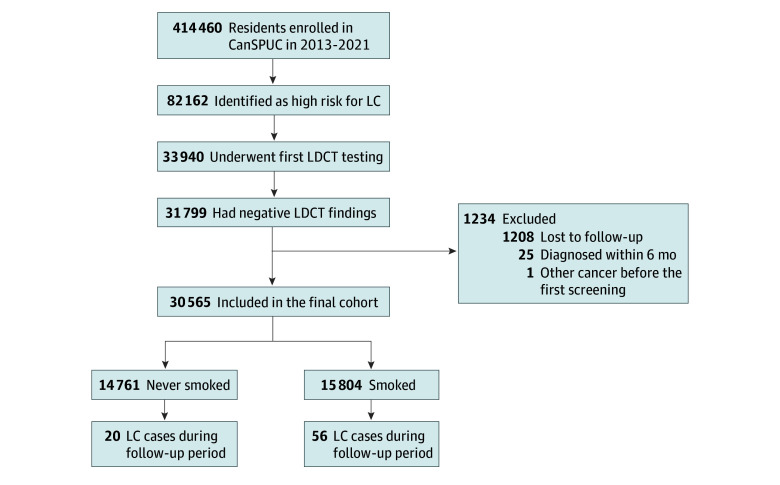
Participant Inclusion and Exclusion Flowchart CanSPUC indicates Cancer Screening Program in Urban China; LC, lung cancer; LDCT, low-dose computed tomography.

Briefly, participants completed a self-administered questionnaire at baseline to evaluate risk factor exposure and quantify cancer risk based on a scoring system derived from the Harvard Cancer Risk Index by the National Cancer Center. Details regarding the scoring system have been published elsewhere.^22^ Only those identified as being at high risk for LC were referred for complimentary LDCT at designated hospitals. LDCT was performed using a 16-slice, multidetector CT scanner (LightSpeed-16, GE Healthcare). According to the CanSPUC protocol,^23^ baseline positive CT findings were defined as (1) solid or partially solid nodules with a mean diameter larger than 5 mm, (2) nonsolid nodules with a diameter larger than 8 mm, (3) tracheal luminal nodules, (4) airway pathology requiring bronchial biopsy, and (5) suspected or confirmed LC. Participants who did not meet any of these positive evaluation criteria were defined as having a negative finding. The participant selection process is outlined in Figure 1. Of the 31 799 individuals with negative baseline LDCT findings, 1208 (3.8%) were unavailable for follow-up, and their baseline characteristics were broadly similar to those retained (eTable 1 in Supplement 1).

This analysis used CanSPUC data from 8 cities in Henan Province (Zhengzhou, Anyang, Luoyang, Nanyang, Zhumadian, Puyang, and Xinxiang) between October 1, 2013, and December 31, 2021. Individuals with negative baseline LDCT findings were included. We also excluded those unavailable for follow-up, with prior cancer, and diagnosed with LC within 6 months of follow-up.

### Definition of Smoking Exposure Parameters

Data on smoking status (never smoker or smoker), pack-years, and time since quitting were collected through self-report at baseline. Smokers were defined as individuals with a history of current or former daily smoking (≥1 cigarette daily for >6 months). Daily cigarette use and smoking duration (years) were recorded. Smoking duration was computed from start to quit age (former smokers) or baseline age (current smokers). Pack-years were calculated as the number of packets (20 cigarettes) per day multiplied by the smoking duration (years). Never smokers were assigned 0 pack-years, whereas smokers were stratified into less than 20, 20 to less than 30, and 30 or more pack-years. For former smokers, time since quitting was categorized as less than 5, 5 to less than 15, or 15 or more years.

### Definition of Covariates

Thirteen potential covariates associated with LC risk were evaluated^13,24^ and categorized into 5 domains: (1) sociodemographic factors: age, sex, educational level, and family history of LC in first-degree relatives (parents or siblings); (2) behavioral factors: physical activity (categorized as heavy [exercise ≥3 d/wk and >90 min/wk] or moderate/none) and alcohol consumption (classified as ever/current [weekly or more for >1 year] or never); (3) history of chronic respiratory diseases: presence (yes) or absence (no) of conditions such as chronic obstructive pulmonary disease, emphysema, silicosis, pneumoconiosis, or tuberculosis; (4) occupational or environmental exposures: occupational exposure to hazardous substances for at least 1 year (no or yes), secondhand smoke exposure (no or yes), solid fuel used for heating in winter (no or yes), solid fuel used for cooking (no or yes), and cooking oil fume exposure (none or a little or a lot); and (5) metabolic risk factors: body mass index (BMI), calculated as weight in kilograms divided by the square of height in meters, with high BMI defined as 24 or greater according to Chinese criteria.^25^ For the occupational or environmental exposure domain, hazardous substances encompassed asbestos, radon, beryllium, uranium, chromium, cadmium, nickel, silicon, diesel exhaust gas, coal smoke, and coal ash. Secondhand smoke was defined as the involuntary inhalation of tobacco smoke in the household or work environment for more than 20 years. Solid fuel included wood, coal, animal dung, and crop waste. Cooking oil fume exposure was categorized based on cooking technology use, with none or a little exposure indicating the use of chimneys, fume extractors, or smokeless pots during cooking, whereas a lot of exposure indicated their absence.

### Follow-Up and Ascertainment of Outcomes

The primary outcome was LC incidence, coded as C34 per the* International Statistical Classification of Diseases and Related Health Problems, Tenth Revision (ICD-10)*. Individuals with negative baseline LDCT findings were not placed under active surveillance within CanSPUC itself. To reflect actual diagnosis patterns after a negative screen result, a combination of passive and active follow-up approaches was implemented.

Passive follow-up was performed by linking data with the Henan Provincial Cancer Registry (HPCR). The HPCR aggregates reports from county-level registries, hospitals, community health centers, and a 3-tier physician network, supplemented by health insurance and vital statistics databases. HPCR data quality adheres to national^26^ and international standards^27^; its detailed procedures have been described elsewhere.^28^ Active follow-up was conducted via telephone or home visits by project physicians, local coordinators, and CanSPUC staff to verify cancer outcomes, including diagnosis date, stage, and cancer-related death. This dual approach ensured capture of all incident LC cases regardless of the clinical context (eg, detected via symptoms, routine health checks, or investigations for other conditions), thereby reflecting actual postscreening diagnostic practice.

### Statistical Analysis

Baseline characteristics were presented as number (percentage) for categorical variables and mean (SD) or median (IQR) for continuous variables. In the estimation of LC incidence, the follow-up person-years were calculated from the baseline LDCT date until the date of LC diagnosis, death, or censoring (December 2023), whichever came first. Cumulative incidence was estimated through Kaplan-Meier method. Multivariable Cox proportional hazards regression models were used to estimate the associations between smoking exposure (status, pack-years, and time since quitting) and LC risk, adjusting for covariates. The proportional hazards assumption was verified globally and for covariates using Schoenfeld residuals (all *P* > .05; no violations detected). Crude and adjusted hazard ratios (AHRs) with 95% CIs were reported. The dose-response association between pack-years and LC risk was examined using restricted cubic splines (RCS) with knots at 0, 20, and 30 pack-years, reflecting current screening guidelines.^12,13,29^

Time-stratified Cox proportional hazards regression models were fitted by annual intervals (years 1-5) to align with screening guidelines and to assess temporal trends in LC risk. Subgroup analyses by sex and age group (40-49, 50-54, and 55-74 years) were conducted to explore association heterogeneity. Time-stratified analyses were not extended to subgroups due to limited statistical power.

Three sensitivity analyses were performed: (1) excluding participants with less than 3 years of follow-up (n = 9610) to reduce subclinical disease bias, (2) accounting for competing risk of all-cause mortality using subdistribution hazard models, and (3) fitting a parsimonious model adjusted only for age, sex, family history of LC in first-degree relatives, and history of chronic respiratory diseases to reduce potential overfitting. All analyses were performed using R software, version 4.3.0 (R Foundation for Statistical Computing). A 2-sided *P* < .05 was considered statistically significant.

## Results

The final cohort included 30 565 individuals (mean [SD] age, 57.1 [7.7] years; 15 693 [51.3%] female), consisting of 14 761 never smokers and 15 804 smokers (1534 former and 14 270 current). Baseline characteristics stratified by smoking status and pack-years are summarized in the Table and eTable 2 in Supplement 1, respectively. Compared with never smokers, smokers were younger and predominantly male, had lower rates of first-degree family history of LC, had higher educational levels, and had higher prevalence of ever or current alcohol use. Smokers also reported higher occupational hazardous exposure, lower chronic respiratory disease prevalence, more solid fuel use for heating and cooking, less cooking oil fume exposure, and higher rates of high BMI.

**Table.** Baseline Characteristics of Study Population by Smoking Status

Characteristic	No. (%) of participants^c^			χ^2^	*P* value
	Never smokers (n = 14 761)	Smokers (n = 15 804)	Total (N = 30 565)		
No. of pack-years					
0	14 761 (100)	NA	14 761 (48.4)	NA	NA
<20	NA	2804 (17.8)	2804 (9.2)		
20-<30	NA	2687 (17.0)	2687 (8.8)		
≥30	NA	10 278 (65.2)	10 278 (33.7)		
Missing	0	35	35		
Time since quitting, y^a^					
<5	NA	845 (55.1)	845 (55.1)	NA	NA
5-<15	NA	555 (36.2)	555 (36.2)		
≥15	NA	134 (8.7)	134 (8.7)		
Median (IQR)	NA	3.0 (1.0-8.0)	3.0 (1.0-8.0)		
Age, y					
40-49	2189 (14.8)	3047 (19.3)	5236 (17.1)	112.41	<.001
50-54	3435 (23.3)	3313 (21.0)	6748 (22.1)		
≥55	9137 (61.9)	9444 (59.8)	18 581 (60.8)		
Mean (SD)	57.3 (7.58)	56.9 (7.94)	57.1 (7.77)		
Sex					
Male	638 (4.3)	14 233 (90.1)	14 871 (48.7)	22 458.09	<.001
Female	14 123 (95.7)	1571 (9.9)	15 694 (51.3)		
Educational level					
Primary school and below	3475 (23.6)	2339 (14.8)	5814 (19.1)	407.07	<.001
Middle or high school	9139 (62.2)	10 654 (67.4)	19 793 (64.9)		
College or above	2081 (14.2)	2811 (17.8)	4892 (16.0)		
Missing	66	0	66		
Family history of lung cancer in first-degree relatives					
No	13 978 (94.7)	15 131 (95.7)	29 109 (95.2)	18.41	<.001
Yes	783 (5.3)	673 (4.3)	1456 (4.8)		
Physical activity					
Moderate or no	10327 (70.3)	11 034 (69.8)	21 361 (70.0)	0.76	.383
Heavy	4368 (29.7)	4770 (30.2)	9138 (30.0)		
Missing	66	0	66		
Alcohol consumption					
Never	12 866 (87.6)	4860 (30.8)	17 726 (58.1)	10 093.55	<.001
Ever or current	1829 (12.4)	10 944 (69.2)	12 773 (41.9)		
Missing	66	0	66		
History of chronic respiratory diseases					
No	5931 (40.4)	7808 (49.4)	13 739 (45.0)	251.63	<.001
Yes	8764 (59.6)	7996 (50.6)	16 760 (55.0)		
Missing	66	0	66		
Occupational exposure to hazardous substances					
No	9322 (63.4)	9294 (58.8)	18 616 (61.0)	68.60	<.001
Yes	5373 (36.6)	6510 (41.2)	11 883 (39.0)		
Missing	66	0	66		
Secondhand smoke exposure^b^					
No	9095 (61.6)	15 804 (100)	24 899 (81.5)	NA	NA
Yes	5666 (38.4)	NA	5666 (18.5)		
Solid fuel used for heating in winter					
No	13 591 (92.1)	13 907 (88.0)	27 498 (90.0)	140.53	<.001
Yes	1170 (7.9)	1897 (12.0)	3067 (10.0)		
Solid fuel used for cooking					
No	14 068 (95.3)	14 597 (92.4)	28 665 (93.8)	113.35	<.001
Yes	693 (4.7)	1207 (7.6)	1900 (6.2)		
Cooking oil fume exposure					
None or a little	596 (4.5)	815 (5.5)	1411 (5.0)	14.35	<.001
A lot	12 676 (95.5)	14 064 (94.5)	26 740 (95.0)		
Missing	1489	925	2414		
High BMI					
No	6511 (44.1)	6156 (39.0)	12 667 (41.4)	83.65	<.001
Yes	8250 (55.9)	9648 (61.0)	17 898 (58.6)		

Abbreviations: BMI, body mass index (calculated as weight in kilograms divided by the square of height in meters); NA, not applicable.

^a^ Information on time since quitting (years) was collected only for former smokers.

^b^ Information on secondhand smoke exposure was collected only for never smokers.

^c^ Unless otherwise indicated.

The median (IQR) follow-up was 4.35 (2.56-6.22) years for the entire cohort, 3.86 (2.26-5.98) years for never smokers, and 4.63 (3.23-6.42) years for smokers. For participants with less than 20, 20 to less than 30, and 30 or ore pack-years, the median (IQR) follow-up was 5.39 (4.19-7.09), 5.10 (4.22-6.95), and 4.34 (2.43-5.91) years, respectively. During a total of 139 011.51 person-years, 76 incident LC cases were identified, yielding a crude incidence rate of 54.67 per 100 000 person-years. The characteristics of LC cases were detailed in eTable 3 in Supplement 1. Among them, 20 occurred in never smokers and 56 in smokers. Never smokers were predominantly female (19 of 20 [95.0%]). Early-stage (stages I-II) diagnoses accounted for 48.2% of cases in smokers and 45.0% in never smokers.

**Figure 2.**
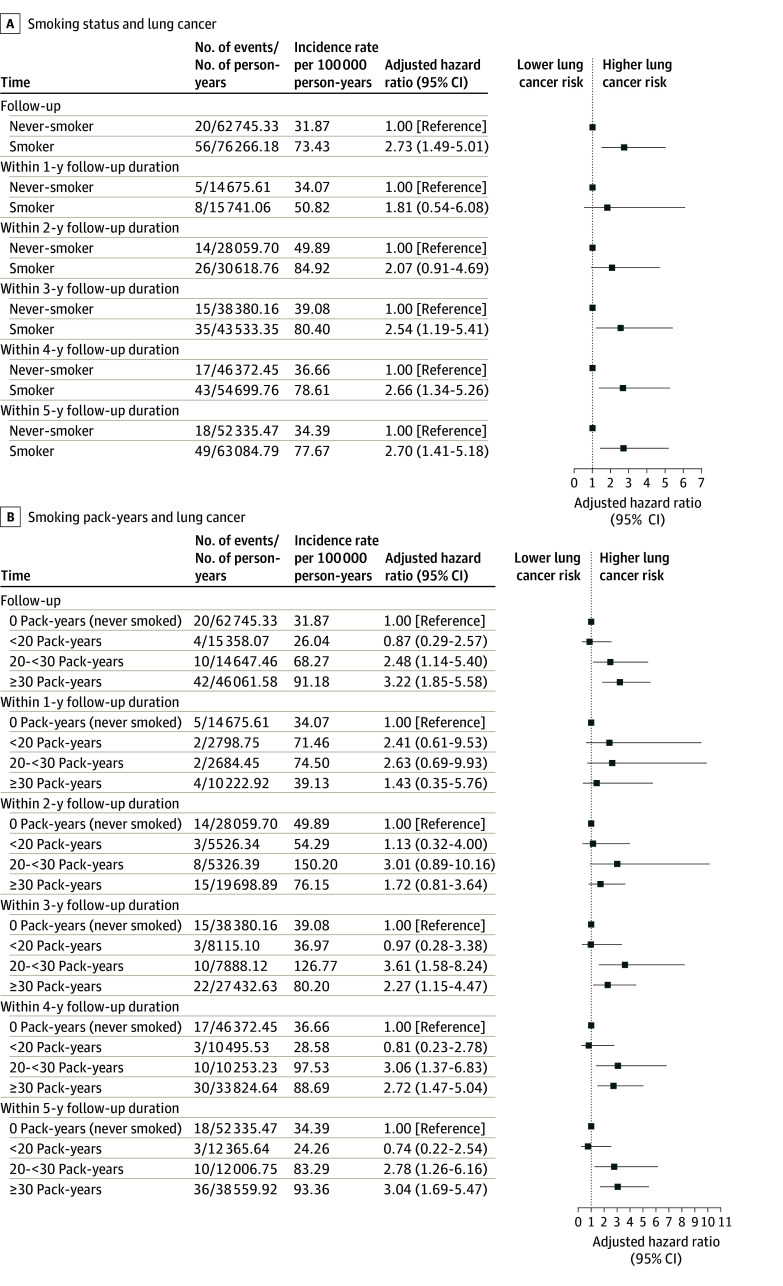
Forest Plot of the Association Between Smoking and Lung Cancer Risk by Follow-Up Duration After Negative Baseline Low-Dose Computed Tomography Findings Error bars indicate 95% CIs.

**Figure 3.**
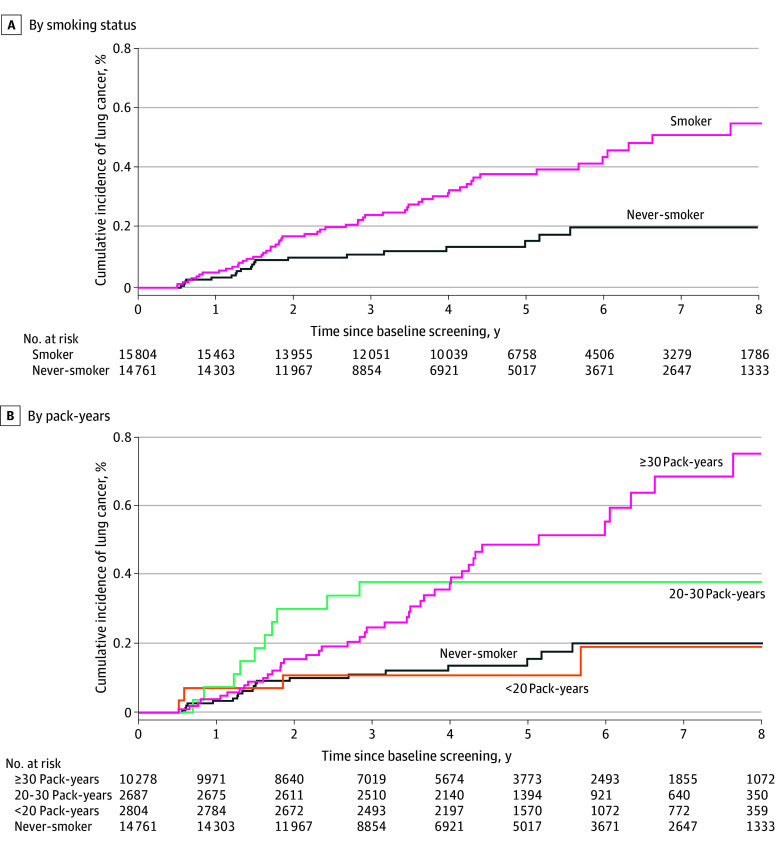
Survival Plots of Cumulative Lung Cancer Incidence After Negative Baseline Low-Dose Computed Tomography Findings

**Figure 4.**
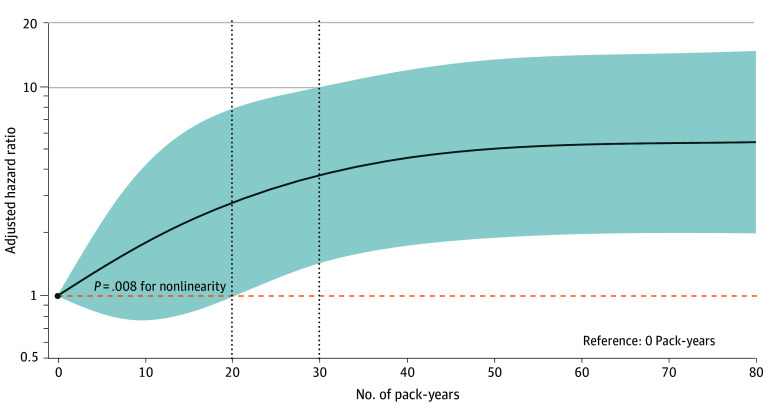
Line Graph of the Dose-Response Association Between Smoking Pack-Years and Lung Cancer Risk Shaded area indicates the 95% CIs; dashed horizontal line, reference hazard ratio of 1; dashed vertical lines, restricted cubic spline knots.

Associations between smoking and LC risk after a negative baseline finding are shown in Figure 2. All covariates satisfied the proportional hazards assumption. After multivariable adjustment, smokers showed a higher LC risk than never smokers (AHR, 2.73; 95% CI, 1.49-5.01). Time-stratified analyses indicated no significant risk increase within the first 2 years (year 1 AHR, 1.81; 95% CI, 0.54-6.08; year 2 AHR, 2.07; 95% CI, 0.91-4.69), but risk increased significantly from year 3 (AHR, 2.54; 95% CI, 1.19-5.41) onward, with HR stabilizing at approximately 2.7 by year 5 (AHR, 2.70; 95% CI, 1.41-5.18) (Figure 2A). When analyzed by pack-years, significantly elevated risks were observed only among those with a smoking history of 20 pack-years or more (20 to <30 pack-years: AHR, 2.48; 95% CI, 1.14-5.40; ≥30 pack-years: AHR, 3.22; 95% CI, 1.85-5.58). Time-stratified patterns were consistent with those for smoking status. Cumulative incidence curves confirmed this pattern, with overlapping curves in the first 2 years and significant divergence thereafter (Figure 3). The RCS analysis revealed a significant nonlinear association between smoking pack-years and LC incidence (χ^2^_3_ = 11.78, *P* = .008), with risk increasing notably beyond 20 pack-years and plateauing at high exposures (Figure 4).

The sex-specific subgroup analysis indicated a stronger association between smoking and LC incidence among females at comparable exposure levels. Specifically, females with a smoking history of 30 pack-years or more had markedly higher risk (AHR, 5.78; 95% CI, 1.87-17.83) than males with similar exposure (AHR, 1.36; 95% CI, 0.18-10.39). Age-specific analysis showed significantly increased risk at a smoking history of 20 pack-years or more in participants aged 55 to 74 years, whereas no statistically significant increased risk was observed at a smoking history of 30 pack-years or more in those aged 50 to 54 years (eTables 4 and 5 in Supplement 1). Sensitivity analyses supported these findings (eTables 6-17 in Supplement 1).

Among 1534 former smokers, 3 LC cases occurred (incidence rate, 43.35 per 100 000 person-years), with all participants having a smoking history of 30 pack-years or more and less than 15 years of cessation. Compared with current smokers, shorter cessation (<15 years) was not associated with significantly reduced LC risk (eTable 18 in Supplement 1). Sensitivity analyses yielded consistent results (eTables 19-21 in Supplement 1).

## Discussion

To our knowledge, this is the first population-based, prospective cohort study to comprehensively characterize the long-term LC risk among individuals with negative baseline LDCT findings, with a specific focus on smoking exposure. We found significantly elevated LC risk in smokers, particularly those with 20 pack-years or more of cumulative tobacco exposure, but this risk was not statistically significant within 2 years after screening. Short-term smoking cessation did not mitigate long-term LC risk among heavily exposed individuals. We also identified nonnegligible residual risk in certain subgroups of never smokers. These findings challenge current screening paradigms and provide actionable evidence for refining LDCT guidelines and tobacco control strategies.

This study also found that a negative baseline LDCT did not eliminate long-term LC risk, underscoring the need for continued surveillance beyond initial screening. In time-stratified analyses, smokers showed no statistically significant elevation in risk within the first 2 years after LDCT, and clinical stage distribution was similar between smokers and never smokers. This temporal pattern aligns with the biological latency of tobacco-induced carcinogenesis in which premalignant lesions likely require several years to develop into radiologically detectable tumors.^30^ These findings suggest that annual screening within the first 2 years offers limited benefit and supports the evaluation of extended screening intervals. In light of the substantial proportion of late-stage diagnoses in our cohort, together with evidence from the NELSON trial that a 2.5-year interval was associated with more late-stage cancers,^31^ a biennial screening interval appears more acceptable than longer intervals to minimize the risk of late-stage diagnosis and mortality. Future cost-effectiveness studies are needed to define the optimal surveillance interval.

RCS analysis revealed a significant nonlinear dose-response association between pack-years and LC incidence, with a clinically relevant threshold of approximately 20 pack-years. This finding empirically supports the USPSTF’s 20–pack-year screening thresholds^12^ while challenging the stringency of China’s 30–pack-years criterion.^13^ Importantly, this threshold was modified by age. Significant risk elevation was observed at 20 pack-years or more in participants aged 55 to 74 years but only at 30 pack-years or more in those aged 50 to 54 years. These findings underscore the value of age-specific risk stratification in screening guidelines to improve efficacy and resource allocation, particularly by potentially lowering the pack-year threshold for older individuals while maintaining higher thresholds for younger age groups.

Although prior studies support the cost-effectiveness of combining screening with smoking cessation,^18,19^ our data suggest that short-term cessation (<15 years) did not significantly reduce LC risk within the available follow-up. The limited number of long-term quitters in our cohort restricts definitive conclusions about risk reduction beyond 15 years. This observation aligns with molecular evidence that heavy smoking induces irreversible genetic damage^32^ and that former smokers may take approximately 22 years to achieve methylation profiles comparable to never smokers.^33^ Prolonged surveillance is thus warranted for former smokers, especially those with substantial smoking history.

Notably, we observed significant sex-based differences in LC risk, with women showing markedly higher AHRs at comparable smoking exposure. The interpretation of this sex differential is limited by the sparse events in the male never smoker reference group (only 1 LC case), which might inflate the apparent greater susceptibility in females. Nevertheless, this observation was consistent with previous reports suggesting greater female vulnerability to tobacco carcinogens.^34,35^ Potential mechanisms may involve estrogen-mediated activation of tobacco carcinogens^36,37^ and X-chromosome–linked DNA repair.^38^ As smoking rates among women in low- and middle-income countries continue to rise,^39^ these findings underscore the need for mechanistic research into sex differences in lung carcinogenesis and support the development of sex-specific screening and cessation programs.

Although LDCT screening is established, concerns regarding false-positive findings, overdiagnosis, and radiation persist.^40^ Current guidelines prioritize heavy smokers to maximize cost-effectiveness.^41,42^ However, our findings reveal a substantial limitation of this smoker-only approach. Strikingly, 19 of 20 LC cases (95%) in never smokers occurred in women, all reporting high cooking fume exposure. This finding aligns with evidence that non–risk-based screening detects cancers in ineligible individuals^43^ and that smoker-focused criteria may underdiagnose many at risk, particularly Chinese women (only 1.0% eligible).^44^ Thus, adopting risk model–based eligibility that incorporates nonsmoking risk factors, rather than relying solely on smoking history, is crucial. Future studies should also explore integrating objective exposure biomarkers to refine stratification.

### Limitations

This study has several limitations. First, although our urban Chinese cohort offers insights for similar high-risk populations, generalizability to rural areas or populations with different smoking patterns may be limited. Second, smoking history and cessation status were self-reported and subject to recall and social desirability biases. Particularly, underreporting in women due to cultural stigma might have influenced sex difference estimates. Future studies could incorporate objective biomarkers (eg, cotinine^45^) to improve exposure assessment. Third, adjusting for multiple covariates, unmeasured confounders, such as air pollution^46^ and genetic factors,^47^ might affect risk estimates. Fourth, limited statistical power in certain analyses, particularly among former smokers and in subgroup comparisons, might have obscured subtle associations. These findings should therefore be considered exploratory and require validation in larger cohorts. Fifth, LC case ascertainment relied on registry data and active follow-up, which might have missed early-stage or asymptomatic cases; however, uniform outcome assessment likely caused nondifferential misclassification, yielding conservative association estimates. Future studies incorporating LDCT rescreening data are warranted.

## Conclusions

This large prospective cohort study found that negative baseline LDCT findings did not provide uniform long-term protection against LC. Heavy smoking (≥20 pack-years) was associated with significantly increased LC risk, with a biologically plausible latency of 3 or more years. Age- and sex-based risk heterogeneity supported the need for personalized screening strategies. The limited risk reduction after short-term cessation underscored the importance of combining smoking cessation programs with long-term LDCT monitoring. The occurrence of LC in never smokers highlighted the need for expanded screening criteria beyond smoking history. Future research should focus on cost-effectiveness analyses of extended screening intervals, long-term follow-up in diverse populations, and mechanistic studies of sex differences to advance precision prevention in individuals with negative LDCT findings.

## Notes

Supplement 1. **eTable 1.** Baseline characteristics between participants lost to follow-up and those retained in the study **eTable 2.** Baseline characteristics of study population by smoking pack-years **eTable 3.** Demographic and clinical characteristics of lung cancer cases **eTable 4.** Association between smoking status and lung cancer risk, by sex and age **eTable 5.** Association between smoking pack-years and lung cancer risk, by sex and age **eTable 6.** Sensitivity analysis of association between smoking status and lung cancer risk, by follow-up duration, after excluding participants with <3 years of follow-up **eTable 7.** Sensitivity analysis of association between smoking status and lung cancer risk, by sex and age, after excluding participants with <3 years of follow-up **eTable 8.** Sensitivity analysis of association between smoking pack-years and lung cancer risk, by follow-up duration, after excluding participants with <3 years of follow-up **eTable 9.** Sensitivity analysis of association between smoking pack-years and lung cancer risk, by sex and age, after excluding participants with <3 years of follow-up **eTable 10.** Sensitivity analysis of association between smoking status and lung cancer risk, by follow-up duration, after accounting for competing risks **eTable 11.** Sensitivity analysis of association between smoking status and lung cancer risk, by sex and age, after accounting for competing risks **eTable 12.** Sensitivity analysis of association between smoking pack-years and lung cancer risk, by follow-up duration, accounting for competing risks **eTable 13.** Sensitivity analysis of association between smoking pack-years and lung cancer risk, by sex and age, after accounting for competing risks **eTable 14.** Sensitivity analysis of association between smoking status and LC incidence risk, by follow-up duration, using a parsimonious model **eTable 15.** Sensitivity analysis of association between smoking status and LC incidence risk, by sex and age, using a parsimonious model **eTable 16.** Sensitivity analysis of association between smoking pack-years and LC incidence risk, by follow-up duration, using a parsimonious model **eTable 17.** Sensitivity analysis of association between smoking pack-years and LC incidence risk, by sex and age, using a parsimonious model **eTable 18.** Association between smoking cessation and lung cancer risk **eTable 19.** Sensitivity analysis of association between smoking cessation and lung cancer risk among smokers, after excluding participants with <3 years of follow-up **eTable 20.** Sensitivity analysis of association between smoking cessation and lung cancer risk among smokers, after accounting for competing risks **eTable 21.** Sensitivity analysis of association between smoking cessation and LC incidence risk, using a parsimonious model

Supplement 2.
Data Sharing Statement
