# Supplementary material for: Tobacco Smoking and Lung Cancer Risk After Negative Baseline Low-Dose Computed Tomography Findings
Source: JAMA Netw Open. 2026 Mar 20;9(3):e261913. doi: 10.1001/jamanetworkopen.2026.1913 (PMC13005165; doi:10.1001/jamanetworkopen.2026.1913)
Supplement: Supplement 1. — eTable 1. Baseline characteristics between participants lost to follow-up and those retained in the study eTable 2. Baseline characteristics of study population by smoking pack-years eTable 3. Demographic and clinical characteristics of lung cancer cases eTable 4. Association between smoking status and lung cancer risk, by sex and age eTable 5. Association between smoking pack-years and lung cancer risk, by sex and age eTable 6. Sensitivity analysis of association between smoking status and lung cancer risk, by follow-up duration, after excluding participants with <3 years of follow-up eTable 7. Sensitivity analysis of association between smoking status and lung cancer risk, by sex and age, after excluding participants with <3 years of follow-up eTable 8. Sensitivity analysis of association between smoking pack-years and lung cancer risk, by follow-up duration, after excluding participants with <3 years of follow-up eTable 9. Sensitivity analysis of association between smoking pack-years and lung cancer risk, by sex and age, after excluding participants with <3 years of follow-up eTable 10. Sensitivity analysis of association between smoking status and lung cancer risk, by follow-up duration, after accounting for competing risks eTable 11. Sensitivity analysis of association between smoking status and lung cancer risk, by sex and age, after accounting for competing risks eTable 12. Sensitivity analysis of association between smoking pack-years and lung cancer risk, by follow-up duration, accounting for competing risks eTable 13. Sensitivity analysis of association between smoking pack-years and lung cancer risk, by sex and age, after accounting for competing risks eTable 14. Sensitivity analysis of association between smoking status and LC incidence risk, by follow-up duration, using a parsimonious model eTable 15. Sensitivity analysis of association between smoking status and LC incidence risk, by sex and age, using a parsimonious model eTable 16. Sensitivity analy [file jamanetwopen-e261913-s001.pdf]

Supplemental Online Content

Liu Y, Guo X, Qie R, et al. Tobacco smoking and lung cancer risk after negative baseline low-dose computed tomography findings. *JAMA Netw Open*. 2026;9(3):e261913. doi:10.1001/jamanetworkopen.2026.1913

eTable 1. Baseline characteristics between participants lost to follow-up and those retained in the study
eTable 2. Baseline characteristics of study population by smoking pack-years
eTable 3. Demographic and clinical characteristics of lung cancer cases
eTable 4. Association between smoking status and lung cancer risk, by sex and age
eTable 5. Association between smoking pack-years and lung cancer risk, by sex and age
eTable 6. Sensitivity analysis of association between smoking status and lung cancer risk, by follow-up duration, after excluding participants with <3 years of follow-up
eTable 7. Sensitivity analysis of association between smoking status and lung cancer risk, by sex and age, after excluding participants with <3 years of follow-up
eTable 8. Sensitivity analysis of association between smoking pack-years and lung cancer risk, by follow-up duration, after excluding participants with <3 years of follow-up
eTable 9. Sensitivity analysis of association between smoking pack-years and lung cancer risk, by sex and age, after excluding participants with <3 years of follow-up
eTable 10. Sensitivity analysis of association between smoking status and lung cancer risk, by follow-up duration, after accounting for competing risks
eTable 11. Sensitivity analysis of association between smoking status and lung cancer risk, by sex and age, after accounting for competing risks
eTable 12. Sensitivity analysis of association between smoking pack-years and lung cancer risk, by follow-up duration, accounting for competing risks
eTable 13. Sensitivity analysis of association between smoking pack-years and lung cancer risk, by sex and age, after accounting for competing risks
eTable 14. Sensitivity analysis of association between smoking status and LC incidence risk, by follow-up duration, using a parsimonious model
eTable 15. Sensitivity analysis of association between smoking status and LC incidence risk, by sex and age, using a parsimonious model
eTable 16. Sensitivity analysis of association between smoking pack-years and LC incidence risk, by follow-up duration, using a parsimonious model
eTable 17. Sensitivity analysis of association between smoking pack-years and LC incidence risk, by sex and age, using a parsimonious model
eTable 18. Association between smoking cessation and lung cancer risk
eTable 19. Sensitivity analysis of association between smoking cessation and lung cancer risk among smokers, after excluding participants with <3 years of follow-up

© 2026 Liu Y et al. *JAMA Network Open*.

eTable 20. Sensitivity analysis of association between smoking cessation and lung cancer risk among smokers, after accounting for competing risks
eTable 21. Sensitivity analysis of association between smoking cessation and LC incidence risk, using a parsimonious model

This supplemental material has been provided by the authors to give readers additional information about their work.

© 2026 Liu Y et al. *JAMA Network Open*.

eTable 1. Baseline characteristics between participants lost to follow-up and those retained in the study

	Lost to follow-up (n=1,208), n(%)	Follow-up (n=30,565), n(%)
**Smoking status**		
Never-smoker	410 (41.2)	14,761 (48.3)
Smoker	584 (58.8)	15,804 (51.7)
Missing	214	0
**Sex**		
Male	528 (53.1)	14,871 (48.7)
Female	466 (46.9)	15,694 (51.3)
**Age**		
40–49	216 (21.7)	5,236 (17.1)
50–54	198 (19.9)	6,748 (22.1)
≥55	580 (58.4)	18,581 (60.8)
**Education level**		
Primary school and below	152 (15.4)	5,814 (19.1)
Middle or high school	676 (68.3)	19,793 (64.9)
College or above	162 (16.4)	4,892 (16.0)
Missing	4	66
**Family history of lung cancer in first-degree relatives**		
No	986 (99.2)	29,109 (95.2)
Yes	8 (0.8)	1,456 (4.8)
**Physical activity**		
Moderate or no	719 (72.6)	21,361 (70.0)
Heavy	271 (27.4)	9,138 (30.0)
Missing	4	66
**Alcohol consumption**		
Never	487 (49.2)	17,726 (58.1)
Ever or current	503 (50.8)	12,773 (41.9)
Missing	4	66
**History of chronic respiratory diseases**		
No	318 (32.1)	13,739 (45.0)
Yes	672 (67.9)	16,760 (55.0)
Missing	4	66
**Occupational exposure to hazardous substances**		
No	534 (53.9)	18,616 (61.0)
Yes	456 (46.1)	11,883 (39.0)
Missing	4	66

© 2026 Liu Y et al. *JAMA Network Open.*

**Secondhand smoke exposure**[#]		
No	964 (97.0)	24,899 (81.5)
Yes	30 (3.0)	5,666 (18.5)
**Solid fuel used for heating in winter**		
No	873 (87.8)	27,498 (90.0)
Yes	121 (12.2)	3,067 (10.0)
**Solid fuel used for cooking**		
No	918 (92.4)	28,665 (93.8)
Yes	76 (7.6)	1,900 (6.2)
**Cooking oil fume exposure**		
None or a little	36 (3.7)	1,411 (5.0)
A lot	939 (96.3)	26,740 (95.0)
Missing	19	2,414
**High BMI**		
No	398 (40.0)	12,667 (41.4)
Yes	596 (60.0)	17,898 (58.6)

[#]Information on secondhand smoke exposure was collected only for never-smokers.

© 2026 Liu Y et al. *JAMA Network Open*.

eTable 2. Baseline characteristics of study population by smoking pack-years

	Never-smoker (n=14,761), n(%)	<20 pack-years (n=2,804), n(%)	20–<30 pack-years (n=2,687), n(%)	≥30 pack-years (n=10,278), n(%)	Total (n=30,530), n(%)
**Time since quitting (years)**[*]					
<5	NA	257 (46.7)	127 (65.1)	455 (58.4)	839 (55.1)
5–<15	NA	194 (35.3)	53 (27.2)	305 (39.2)	552 (36.2)
≥15	NA	99 (18.0)	15 (7.7)	19 (2.4)	133 (8.7)
Median (IQR)	NA	5.0 (1.0,10.0)	1.0 (1.0,6.0)	3.0 (1.0,6.0)	3.0 (1.0,8.0)
**Age**					
40–49	2,189 (14.8)	905 (32.3)	946 (35.2)	1,188 (11.6)	5,228 (17.1)
50–54	3,435 (23.3)	620 (22.1)	520 (19.4)	2,168 (21.1)	6,743 (22.1)
≥55	9,137 (61.9)	1279 (45.6)	1221 (45.4)	6,922 (67.3)	18,559 (60.8)
Mean±SD	57.3±7.58	54.3±8.17	54.3±8.38	58.3±7.38	57.1±7.77
**Sex**					
Male	638 (4.3)	1,950 (69.5)	2,478 (92.2)	9,787 (95.2)	14,853 (48.7)
Female	14,123 (95.7)	854 (30.5)	209 (7.8)	491 (4.8)	15,677 (51.3)
**Education level**					
Primary school and below	3,475 (23.6)	298 (10.6)	309 (11.5)	1,725 (16.8)	5,807 (19.1)
Middle or high school	9,139 (62.2)	1,895 (67.6)	1,794 (66.8)	6,944 (67.6)	19,772 (64.9)
College or above	2,081 (14.2)	611 (21.8)	584 (21.7)	1,609 (15.7)	4,885 (16.0)
Missing	66	0	0	0	66
**Family history of lung cancer in first-degree relatives**					
No	13,978 (94.7)	2,753 (98.2)	2,662 (99.1)	9,681 (94.2)	29,074 (95.2)
Yes	783 (5.3)	51 (1.8)	25 (0.9)	597 (5.8)	1,456 (4.8)
**Physical activity**					
Moderate or no	10,327 (70.3)	2,181 (77.8)	1,720 (64.0)	7,106 (69.1)	21,334 (70.0)
Heavy	4,368 (29.7)	623 (22.2)	967 (36.0)	3,172 (30.9)	9,130 (30.0)
Missing	66	0	0	0	66
**Alcohol consumption**					
Never	12,866 (87.6)	893 (31.8)	712 (26.5)	3,240 (31.5)	17,711 (58.1)
Ever or current	1,829 (12.4)	1,911 (68.2)	1,975 (73.5)	7,038 (68.5)	12,753 (41.9)
Missing	66	0	0	0	66
**History of chronic respiratory diseases**					
No	5,931 (40.4)	1,056 (37.7)	1,445 (53.8)	5,305 (51.6)	13,737 (45.1)
Yes	8,764 (59.6)	1,748 (62.3)	1,242 (46.2)	4,973 (48.4)	16,727 (54.9)
Missing	66	0	0	0	66
**Occupational exposure to hazardous substances**					
No	9,322 (63.4)	1,321 (47.1)	1,524 (56.7)	6,435 (62.6)	18,602 (61.1)

© 2026 Liu Y et al. *JAMA Network Open.*

Yes	5,373 (36.6)	1,483 (52.9)	1,163 (43.3)	3,843 (37.4)	11,862 (38.9)
Missing	66	0	0	0	66
**Secondhand smoke exposure**[#]					
No	9,095 (61.6)	2,804 (100)	2,687 (100)	10,278 (100)	24,864 (81.4)
Yes	5,666 (38.4)	NA	NA	NA	5,666 (18.6)
**Solid fuel used for heating in winter**					
No	13,591 (92.1)	2,409 (85.9)	2,329 (86.7)	9,139 (88.9)	27,468 (90.0)
Yes	1,170 (7.9)	395 (14.1)	358 (13.3)	1139 (11.1)	3,062 (10.0)
**Solid fuel used for cooking**					
No	14,068 (95.3)	2,549 (90.9)	2,451 (91.2)	9,568 (93.1)	28,636 (93.8)
Yes	693 (4.7)	255 (9.1)	236 (8.8)	710 (6.9)	1,894 (6.2)
**Cooking oil fume exposure**					
None or a little	596 (4.5)	147 (5.3)	170 (6.4)	496 (5.2)	1,409 (5.0)
A lot	12,676 (95.5)	2,605 (94.7)	2,473 (93.6)	8,955 (94.8)	26,709 (95.0)
Missing	1489	52	44	827	2412
**High BMI**					
No	6,511 (44.1)	1,100 (39.2)	1,034 (38.5)	4,009 (39.0)	12,654 (41.4)
Yes	8,250 (55.9)	1,704 (60.8)	1,653 (61.5)	6,269 (61.0)	17,876 (58.6)

35 participants did not report smoking pack-years.

[*]Information on time since quitting (years) was collected only for former smokers.

[#]Information on secondhand smoke exposure was collected only for never–smokers.

© 2026 Liu Y et al. *JAMA Network Open*.

eTable 3. Demographic and clinical characteristics of lung cancer cases

	Never-smoker (n=20), n(%)	Smoker (n=56), n(%)	All cases (n=76), n(%)
**Smoking pack-years**			
0	20 (100)	NA	20 (26.3)
<20	NA	4 (7.1)	4 (5.3)
20–<30	NA	10 (17.9)	10 (13.2)
≥30	NA	42 (75.0)	42 (55.3)
**Time since quitting (years)***			
<5	NA	1 (33.3)	1 (33.3)
5–<15	NA	2 (66.7)	2 (66.7)
≥15	NA	0 (0)	0 (0)
Median (IQR)	NA	5.0 (2.0, 5.0)	5.0 (2.0, 5.0)
**Age**			
40–49	1 (5.0)	4 (7.1)	5 (6.6)
50–54	5 (25.0)	9 (16.1)	14 (18.4)
55–74	14 (70.0)	43 (76.8)	57 (75.0)
Mean±SD	59.6±7.80	61.8±7.77	61.2±7.79
**Sex**			
Male	1 (5.0)	50 (89.3)	51 (67.1)
Female	19 (95.0)	6 (10.7)	25 (32.9)
**Education level**			
Primary school and below	3 (15.0)	10 (17.9)	13 (17.1)
Middle or high school	14 (70.0)	39 (69.6)	53 (69.7)
College or above	3 (15.0)	7 (12.5)	10 (13.2)
**Family history of lung cancer in first-degree relatives**			
No	19 (95.0)	54 (96.4)	73 (96.1)
Yes	1 (5.0)	2 (3.6)	3 (3.9)
**Physical activity**			
Moderate or no	16 (80.0)	33 (58.9)	49 (64.5)
Heavy	4 (20.0)	23 (41.1)	27 (35.5)
**Alcohol consumption**			
Never	13 (65.0)	16 (28.6)	29 (38.2)
Ever or current	7 (35.0)	40 (71.4)	47 (61.8)
**History of chronic respiratory diseases**			
No	3 (15.0)	21 (37.5)	24 (31.6)
Yes	17 (85.0)	35 (62.5)	52 (68.4)
**Occupational exposure to hazardous substances**			
No	10 (50.0)	28 (50.0)	38 (50.0)
Yes	10 (50.0)	28 (50.0)	38 (50.0)

© 2026 Liu Y et al. *JAMA Network Open.*

**Secondhand smoke exposure[#]**			
No	14 (70.0)	56 (100)	70 (92.1)
Yes	6 (30.0)		6 (7.9)
**Solid fuel used for heating in winter**			
No	18 (90.0)	45 (80.4)	63 (82.9)
Yes	2 (10.0)	11 (19.6)	13 (17.1)
**Solid fuel used for cooking**			
No	19 (95.0)	54 (96.4)	73 (96.1)
Yes	1 (5.0)	2 (3.6)	3 (3.9)
**Cooking oil fume exposure**			
None or a little	1 (5.0)	3 (5.4)	4 (5.3)
A lot	19 (95.0)	53 (94.6)	71 (94.7)
**High BMI**			
No	13 (65.0)	28 (50.0)	41 (53.9)
Yes	7 (35.0)	28 (50.0)	35 (46.1)
**Distribution of clinical stage at diagnosis**			
I	1 (5.0)	4 (7.1)	5 (6.6)
II	8 (40.0)	23 (41.1)	31 (40.8)
III	10 (50.0)	25 (44.6)	35 (46.0)
IV	1 (5.0)	4 (7.1)	5 (6.6)

NA=not applicable.

[*]Information on time since quitting (years) was collected only for formerly smoker.

[#]Information on secondhand smoke exposure was collected only for never-smoker.

© 2026 Liu Y et al. *JAMA Network Open*.

eTable 4. Association between smoking status and lung cancer risk, by sex and age

	Events	Person-years	Incidence rate per 100000 person-years	Crude HR (95%CI)	Adjusted HR* (95%CI)
**By sex**					
**Male**					
Never-smoker	1	1,696.89	58.93	Ref	Ref
Smoker	50	67,028.04	74.60	1.28 (0.18–9.36)	1.16 (0.15–8.78)
**Female**					
Never-smoker	19	61,048.44	31.12	Ref	Ref
Smoker	6	9,238.13	64.95	2.27 (0.90–5.72)	1.28 (0.44–3.75)
**By age, year**					
**40–49**					
Never-smoker	1	13,409.76	7.46	Ref	Ref
Smoker	4	18,053.14	22.16	2.88 (0.32–25.73)	2.51 (0.26–24.68)
**50–54**					
Never-smoker	5	14,251.89	35.08	Ref	Ref
Smoker	9	16,157.13	55.70	1.58 (0.46–5.41)	1.80 (0.59–5.48)
**55–74**					
Never-smoker	14	35,083.68	39.90	Ref	Ref
Smoker	43	42,055.91	102.24	2.57 (1.41–4.71)	2.23 (1.01–4.91)

lung cancer=lung cancer; HR=hazard ratio; CI=confidence interval; Ref=reference. *Adjusted for age/sex, education level, family history of lung cancer in first-degree relatives, physical activity, alcohol consumption, history of chronic respiratory diseases, occupational exposure to hazardous substances, secondhand smoke exposure, solid fuel used for heating in winter, solid fuel used for cooking, cooking oil fume exposure, and high BMI.

© 2026 Liu Y et al. *JAMA Network Open*.

eTable 5. Association between smoking pack-years and lung cancer risk, by sex and age

	Events	Person-years	Incidence rate per 100000 person-years	Crude HR (95%CI)	Adjusted HR* (95%CI)
**By sex**					
**Male**					
0 pack-years (Never-smoker)	1	1,696.89	58.93	Ref	Ref
<20 pack-years	3	10,071.73	29.79	0.51 (0.05–4.92)	0.43 (0.04–4.35)
20–<30 pack-years	9	13,397.69	67.18	1.15 (0.14–9.11)	1.06 (0.13–8.77)
≥30 pack-years	38	43,447.45	87.46	1.50 (0.20–10.97)	1.36 (0.18–10.39)
**Female**					
0 pack-years (Never-smoker)	19	61,048.44	31.12	Ref	Ref
<20 pack-years	1	5,286.34	18.92	0.67 (0.09–5.01)	0.70 (0.09–5.36)
20–<30 pack-years	1	1,249.77	80.01	2.82 (0.38–21.11)	2.93 (0.38–22.43)
≥30 pack-years	4	2,614.13	153.01	5.19 (1.76–15.30)	5.78 (1.87–17.83)
**By age, year**					
**40–49**					
0 pack-years (Never-smoker)	1	13,409.76	7.46	Ref	Ref
<20 pack-years	0	5,515.53	0	NA	NA
20–<30 pack-years	3	5,405.17	55.50	7.00 (0.73–67.31)	6.77 (0.62–74.41)
≥30 pack-years	1	7,078.17	14.13	1.84 (0.11–29.39)	1.71 (0.10–29.30)
**50–54**					
0 pack-years (Never-smoker)	5	14251.89	35.08	Ref	Ref
<20 pack-years	0	3410.7315	0	NA	NA
20–<30 pack-years	0	2914.3123	0	NA	NA
≥30 pack-years	9	9802.3945	91.81	2.64 (0.88–7.89)	2.97 (1.01–8.73)
**55–74**					
0 pack-years (Never-smoker)	14	35,083.68	39.90	Ref	Ref
<20 pack-years	4	6,431.80	62.19	1.56 (0.51–4.75)	1.58 (0.52–4.79)
20–<30 pack-years	7	6,327.98	110.62	2.78 (1.12–6.91)	2.75 (1.11–6.82)
≥30 pack-years	32	29,181.01	109.66	2.76 (1.47–5.17)	2.74 (1.46–5.14)

HR=hazard ratio; CI=confidence interval; Ref=reference; NA=not applicable. *Adjusted for age/sex, education level, family history of lung cancer in first-degree relatives, physical activity, alcohol consumption, history of chronic respiratory diseases, occupational exposure to hazardous substances, secondhand smoke exposure, solid fuel used for heating in winter, solid fuel used for cooking, cooking oil fume exposure, and high BMI.

© 2026 Liu Y et al. *JAMA Network Open*.

eTable 6. Sensitivity analysis of association between smoking status and lung cancer risk, by follow-up duration, after excluding participants with <3 years of follow-up

	Events	Person-years	Incidence rate per 100000 person-years	Crude HR (95%CI)	Adjusted HR* (95%CI)
**Entire follow-up period**					
Never-smoker	20	62,745.33	31.87	Ref	Ref
Smoker	56	76,266.18	73.43	2.33 (1.40–3.88)	2.73 (1.49–5.01)
**Within 1-year follow-up duration**					
Never-smoker	5	14,675.61	34.07	Ref	Ref
Smoker	8	15,741.06	50.82	1.49 (0.49–4.56)	2.39 (0.49–11.77)
**Within 2-year follow-up duration**					
Never-smoker	14	28,059.70	49.89	Ref	Ref
Smoker	26	30,618.76	84.92	1.69 (0.88–3.23)	2.07 (0.91–4.69)
**Within 3-year follow-up duration**					
Never-smoker	15	38,380.16	39.08	Ref	Ref
Smoker	35	43,533.35	80.40	2.06 (1.13–3.77)	2.54 (1.19–5.41)
**Within 4-year follow-up duration**					
Never-smoker	17	46,372.45	36.66	Ref	Ref
Smoker	43	54,699.76	78.61	2.16 (1.23–3.78)	2.66 (1.34–5.26)
**Within 5-year follow-up duration**					
Never-smoker	18	52,335.47	34.39	Ref	Ref
Smoker	49	63,084.79	77.67	2.28 (1.33–3.91)	2.70 (1.41–5.18)

HR=hazard ratio; CI=confidence interval; Ref=reference. *Adjusted for age, sex, education level, family history of lung cancer in first-degree relatives, physical activity, alcohol consumption, history of chronic respiratory diseases, occupational exposure to hazardous substances, secondhand smoke exposure, solid fuel used for heating in winter, solid fuel used for cooking, cooking oil fume exposure, and high BMI.

© 2026 Liu Y et al. *JAMA Network Open*.

eTable 7. Sensitivity analysis of association between smoking status and lung cancer risk, by sex and age, after excluding participants with <3 years of follow-up

	Events	Person-years	Incidence rate per 100000 person-years	Crude HR (95%CI)	Adjusted HR* (95%CI)
**By sex**					
**Male**					
Never-smoker	1	861.32	116.10	Ref	Ref
Smoker	50	59,984.46	83.35	0.76 (0.10–5.50)	0.68 (0.08–5.15)
**Female**					
Never-smoker	19	50,084.84	37.94	Ref	Ref
Smoker	6	8,957.76	66.98	1.82 (0.73–4.57)	1.059 (0.38–2.94)
**By age, year**					
**40–49**					
Never-smoker	1	13,357.29	7.49	Ref	Ref
Smoker	4	18,028.88	22.19	2.87 (0.32–25.65)	1.74 (0.13–22.83)
**50–54**					
Never-smoker	5	11,392.04	43.89	Ref	Ref
Smoker	9	14,682.93	61.30	1.39 (0.47–4.16)	1.84 (0.43–7.82)
**55–74**					
Never-smoker	14	26,196.83	53.44	Ref	Ref
Smoker	43	36,230.41	118.68	2.22 (1.21–4.05)	2.17 (1.18–3.99)

HR=hazard ratio; CI=confidence interval; Ref=reference. *Adjusted for age/sex, education level, family history of lung cancer in first-degree relatives, physical activity, alcohol consumption, history of chronic respiratory diseases, occupational exposure to hazardous substances, secondhand smoke exposure, solid fuel used for heating in winter, solid fuel used for cooking, cooking oil fume exposure, and high BMI.

© 2026 Liu Y et al. *JAMA Network Open.*

eTable 8. Sensitivity analysis of association between smoking pack-years and lung cancer risk, by follow-up duration, after excluding participants with <3 years of follow-up

	Events	Person-years	Incidence rate per 100000 person-years	Crude HR (95%CI)	Adjusted HR* (95%CI)
**Entire follow-up period**					
0 pack-years (Never-smoker)	20	50,946.16	39.26	Ref	Ref
<20 pack-years	4	14,724.91	27.16	0.69 (0.24–2.03)	0.94 (0.31–2.87)
20–<30 pack-years	10	14,305.23	69.90	1.76 (0.82–3.76)	2.47 (1.09–5.57)
≥30 pack-years	42	39,723.52	105.73	2.67 (1.57–4.55)	3.64 (1.98–6.67)
**Within 1-year follow-up duration**					
0 pack-years (Never-smoker)	5	8,867.34	56.39	Ref	Ref
<20 pack-years	2	2,495.09	80.16	1.42 (0.28–7.33)	3.06 (0.43–21.82)
20–<30 pack-years	2	2,519.53	79.38	1.41 (0.27–7.26)	3.13 (0.44–22.33)
≥30 pack-years	4	7,039.71	56.82	1.01 (0.27–3.75)	2.19 (0.40–11.97)
**Within 2-year follow-up duration**					
0 pack-years (Never-smoker)	14	17,726.30	78.98	Ref	Ref
<20 pack-years	3	4,988.94	60.13	0.76 (0.22–2.65)	0.85 (0.24–2.98)
20–<30 pack-years	8	5,034.64	158.90	2.01 (0.84–4.80)	2.24 (0.923–5.41)
≥30 pack-years	15	14,071.41	106.60	1.35 (0.65–2.80)	1.44 (0.69–3.04)
**Within 3-year follow-up duration**					
0 pack-years (Never-smoker)	15	26,580.99	56.43	Ref	Ref
<20 pack-years	3	7,481.94	40.10	0.71 (0.21–2.45)	0.81 (0.23–2.83)
20–<30 pack-years	10	7,545.89	132.52	2.35 (1.05–5.23)	2.71 (1.20–6.12)
≥30 pack-years	22	21,094.56	104.29	1.85 (0.96–3.56)	2.00 (1.02–3.92)
**Within 4-year follow-up duration**					
0 pack-years (Never-smoker)	17	34,573.28	49.17	Ref	Ref
<20 pack-years	3	9,862.37	30.42	0.62 (0.18–2.11)	0.90 (0.25–3.23)
20–<30 pack-years	10	9,911.00	100.90	2.05 (0.94–4.48)	3.07 (1.30–7.24)
≥30 pack-years	30	27,486.58	109.14	2.22 (1.22–4.02)	3.15 (1.58–6.29)
**Within 5-year follow-up duration**					
0 pack-years (Never-smoker)	18	40,536.30	44.40	Ref	Ref
<20 pack-years	3	11,732.48	25.57	0.58 (0.17–1.96)	0.82 (0.23–2.91)
20–<30 pack-years	10	11,664.52	85.73	1.93 (0.89–4.18)	2.81 (1.21–6.52)
≥30 pack-years	36	32,221.85	111.73	2.51 (1.43–4.42)	3.53 (1.84–6.80)

HR=hazard ratio; CI=confidence interval; Ref=reference. *Adjusted for age, sex, education level, family history of lung cancer in first–degree relatives, physical activity, alcohol consumption, history of chronic respiratory diseases, occupational exposure to hazardous substances, secondhand smoke exposure, solid fuel used for heating in winter, solid fuel used for cooking, cooking oil fume exposure, and high BMI.

© 2026 Liu Y et al. *JAMA Network Open*.

eTable 9. Sensitivity analysis of association between smoking pack-years and lung cancer risk, by sex and age, after excluding participants with <3 years of follow-up

	Events	Person-years	Incidence rate per 100000 person-years	Crude HR (95%CI)	Adjusted HR* (95%CI)
**By sex**					
**Male**					
0 pack-years (Never-smoker)	1	861.32	116.10	Ref	Ref
<20 pack-years	3	9,516.71	31.52	0.29 (0.03–2.75)	0.21 (0.02–2.08)
20–<30 pack-years	9	13,084.11	68.79	0.62 (0.08–4.92)	0.46 (0.06–3.71)
≥30 pack-years	38	37,275.59	101.94	0.93 (0.13–6.79)	0.67 (0.09–4.96)
**Female**					
0 pack-years (Never-smoker)	19	50,084.84	37.94	Ref	Ref
<20 pack-years	1	5,208.20	19.20	0.53 (0.07–3.93)	0.55 (0.07–4.17)
20–<30 pack-years	1	1,221.12	81.89	2.23 (0.30–16.69)	2.37 (0.31–17.84)
≥30 pack-years	4	2,447.92	163.40	4.38 (1.49–12.88)	5.00 (1.68–14.88)
**By age, year**					
**40–49**					
0 pack-years (Never-smoker)	1	13,357.29	7.49	Ref	Ref
<20 pack-years	0	5,497.98	0	NA	NA
20–<30 pack-years	3	5,400.95	55.55	6.97 (0.72–67.05)	7.47 (0.77–72.43)
≥30 pack-years	1	7,075.69	14.13	1.83 (0.11–29.26)	1.71 (0.11–27.67)
**50–54**					
0 pack-years (Never-smoker)	5	11,392.04	43.89	Ref	Ref
<20 pack-years	0	3,299.68	0	NA	NA
20–<30 pack-years	0	2,844.93	0	NA	NA
≥30 pack-years	9	8,509.61	105.76	2.40 (0.80–7.16)	3.17 (1.15–8.74)
**55–74**					
0 pack-years (Never-smoker)	14	26,196.83	53.44	Ref	Ref
<20 pack-years	4	5,927.25	67.48	0.58 (0.17–1.97)	0.63 (0.18–2.17)
20–<30 pack-years	7	6,059.35	115.52	1.93 (0.89–4.18)	2.16 (0.99–4.73)
≥30 pack-years	32	24,138.22	132.57	2.51 (1.43–4.42)	2.71 (1.52–4.83)

HR=hazard ratio; CI=confidence interval; Ref=reference; NA=not applicable. *Adjusted for age/sex, education level, family history of lung cancer in first-degree relatives, physical activity, alcohol consumption, history of chronic respiratory diseases, occupational exposure to hazardous substances, secondhand smoke exposure, solid fuel used for heating in winter, solid fuel used for cooking, cooking oil fume exposure, and high BMI.

© 2026 Liu Y et al. *JAMA Network Open*.

eTable 10. Sensitivity analysis of association between smoking status and lung cancer risk, by follow-up duration, after accounting for competing risks

	Events	Person-years	Crude HR (95%CI)	Adjusted HR* (95%CI)
**Entire follow-up period**				
Never-smoker	20	62,745.33	Ref	Ref
Smoker	56	76,266.18	2.32 (1.40–3.86)	2.73 (1.49–5.01)
**Within 1-year follow-up duration**				
Never-smoker	5	14,675.61	Ref	Ref
Smoker	8	15,741.06	1.49 (0.49–4.56)	1.81 (0.54–6.07)
**Within 2-year follow-up duration**				
Never-smoker	14	28,059.70	Ref	Ref
Smoker	26	30,618.76	1.69 (0.88–3.23)	2.07 (0.91–4.71)
**Within 3-year follow-up duration**				
Never-smoker	15	38,380.16	Ref	Ref
Smoker	35	43,533.35	2.06 (1.13–3.76)	2.54 (1.19–5.41)
**Within 4-year follow-up duration**				
Never-smoker	17	46,372.45	Ref	Ref
Smoker	43	54,699.76	2.16 (1.23–3.77)	2.62 (1.31–5.22)
**Within 5-year follow-up duration**				
Never-smoker	18	52,335.47	Ref	Ref
Smoker	49	63,084.79	2.28 (1.33–3.89)	2.70 (1.40–5.20)

HR=hazard ratio; CI=confidence interval; Ref=reference. *Adjusted for age, sex, education level, family history of lung cancer in first-degree relatives, physical activity, alcohol consumption, history of chronic respiratory diseases, occupational exposure to hazardous substances, secondhand smoke exposure, solid fuel used for heating in winter, solid fuel used for cooking, cooking oil fume exposure, and high BMI.

© 2026 Liu Y et al. *JAMA Network Open*.

eTable 11. Sensitivity analysis of association between smoking status and lung cancer risk, by sex and age, after accounting for competing risks

	Events	Person-years	Crude HR (95%CI)	Adjusted HR* (95%CI)
**By sex**				
**Male**				
Never-smoker	1	1,696.89	Ref	Ref
Smoker	50	67,028.04	1.29 (0.180–9.24)	1.16 (0.16–8.59)
**Female**				
Never-smoker	19	61,048.44	Ref	Ref
Smoker	6	9,238.13	2.27 (0.90–5.73)	1.28 (0.46–3.53)
**By age, year**				
**40–49**				
Never-smoker	1	13,409.76	Ref	Ref
Smoker	4	18,053.14	2.87 (0.32–25.79)	2.51 (0.27–22.90)
**50–54**				
Never-smoker	5	14,251.89	Ref	Ref
Smoker	9	16,157.13	1.623 (0.57–4.65)	1.80 (0.69–4.70)
**55–74**				
Never-smoker	14	35,083.68	Ref	Ref
Smoker	43	42,055.91	2.57 (1.40–4.70)	2.23 (0.93–5.35)

HR=hazard ratio; CI=confidence interval; Ref=reference. *Adjusted for age/sex, education level, family history of lung cancer in first-degree relatives, physical activity, alcohol consumption, history of chronic respiratory diseases, occupational exposure to hazardous substances, secondhand smoke exposure, solid fuel used for heating in winter, solid fuel used for cooking, cooking oil fume exposure, and high BMI.

© 2026 Liu Y et al. *JAMA Network Open*.

eTable 12. Sensitivity analysis of association between smoking pack-years and lung cancer risk, by follow-up duration, accounting for competing risks

	Events	Person-years	Crude HR (95%CI)	Adjusted HR* (95%CI)
**Entire follow-up period**				
0 pack-years (Never-smoker)	20	62,745.33	Ref	Ref
<20 pack-years	4	15,358.07	0.83 (0.28–2.45)	0.87 (0.29–2.57)
20–<30 pack-years	10	14,647.46	2.17 (1.01–4.67)	2.47 (1.15–5.34)
≥30 pack-years	42	46,061.58	2.86 (1.69–4.86)	3.21 (1.86–5.55)
**Within 1-year follow-up duration**				
0 pack-years (Never-smoker)	5	14,675.61	Ref	Ref
<20 pack-years	2	2,798.75	2.10 (0.41–10.82)	2.41 (0.61–9.59)
20–<30 pack-years	2	2,684.45	2.18 (0.42–11.25)	2.63 (0.67–10.30)
≥30 pack-years	4	10,222.92	1.15 (0.31–4.27)	1.43 (0.36–5.57)
**Within 2-year follow-up duration**				
0 pack-years (Never-smoker)	14	28,059.70	Ref	Ref
<20 pack-years	3	5,526.34	1.07 (0.31–3.74)	1.13 (0.32–4.03)
20–<30 pack-years	8	5,326.39	2.97 (0.93–9.53)	3.01 (0.89–10.10)
≥30 pack-years	15	19,698.89	1.52 (0.73–3.15)	1.72 (0.83–3.57)
**Within 3-year follow-up duration**				
0 pack-years (Never-smoker)	15	38,380.16	Ref	Ref
<20 pack-years	3	8,115.10	0.95 (0.27–3.29)	0.97 (0.27–3.40)
20–<30 pack-years	10	7,888.12	3.26 (1.47–7.24)	3.60 (1.61–8.04)
≥30 pack-years	22	27,432.63	2.05 (1.07–3.95)	2.27 (1.17–4.41)
**Within 4-year follow-up duration**				
0 pack-years (Never-smoker)	17	46,372.45	Ref	Ref
<20 pack-years	3	10,495.53	0.79 (0.23–2.71)	0.81 (0.23–2.80)
20–<30 pack-years	10	10,253.23	2.69 (1.23–5.90)	3.05 (1.39–6.71)
≥30 pack-years	30	33,824.64	2.42 (1.34–4.38)	2.72 (1.48–5.00)
**Within 5-year follow-up duration**				
0 pack-years (Never-smoker)	18	52,335.47	Ref	Ref
<20 pack-years	3	12,365.64	0.72 (0.21–2.45)	0.74 (0.21–2.56)
20–<30 pack-years	10	12,006.75	2.45 (1.13–5.35)	2.78 (1.27–6.07)
≥30 pack-years	36	38,559.92	2.72 (1.55–4.77)	3.04 (1.71–5.41)

HR=hazard ratio; CI=confidence interval; Ref=reference. *Adjusted for age, sex, education level, family history of lung cancer in first-degree relatives, physical activity, alcohol consumption, history of chronic respiratory diseases, occupational exposure to hazardous substances, secondhand smoke exposure, solid fuel used for heating in winter, solid fuel used for cooking, cooking oil fume exposure, and high BMI.

© 2026 Liu Y et al. *JAMA Network Open*.

eTable 13. Sensitivity analysis of association between smoking pack-years and lung cancer risk, by sex and age, after accounting for competing risks

	Events	Person-years	Crude HR (95%CI)	Adjusted HR* (95%CI)
**By sex**				
**Male**				
0 pack-years (Never-smoker)	1	1,696.89	Ref	Ref
<20 pack-years	3	10,071.73	0.51 (0.05–4.99)	0.44 (0.04–4.52)
20–<30 pack-years	9	13,397.69	1.14 (0.14–9.17)	1.05 (0.12–8.94)
≥30 pack-years	38	43,447.45	1.50 (0.21–10.79)	1.36 (0.19–9.93)
**Female**				
0 pack-years (Never-smoker)	19	61,048.44	Ref	Ref
<20 pack-years	1	5,286.34	0.67 (0.09–4.81)	0.70 (0.09–5.36)
20–<30 pack-years	1	1,249.77	2.82 (0.37–21.37)	2.93 (0.38–22.43)
≥30 pack-years	4	2,614.13	5.20 (1.75–15.42)	5.78 (1.87–17.83)
**By age, year**				
**40–49**				
0 pack-years (Never-smoker)	1	13,409.76	Ref	Ref
<20 pack-years	0	5,515.53	NA	NA
20–<30 pack-years	3	5,405.17	6.99 (0.73–67.26)	6.77 (0.52–88.46)
≥30 pack-years	1	7,078.17	1.84 (0.11–29.55)	1.71 (0.14–20.71)
**50–54**				
0 pack-years (Never-smoker)	5	14251.89	Ref	Ref
<20 pack-years	0	3410.7315	NA	NA
20–<30 pack-years	0	2914.3123	NA	NA
≥30 pack-years	9	9802.3945	2.64 (0.91–7.70)	2.88 (1.08–7.64)
**55–74**				
0 pack-years (Never-smoker)	14	35,083.68	Ref	Ref
<20 pack-years	4	6,431.80	1.56 (0.51–4.79)	1.57 (0.51–4.83)
20–<30 pack-years	7	6,327.98	2.78 (1.10–7.01)	2.75 (1.09–6.93)
≥30 pack-years	32	29,181.01	2.75 (1.47–5.15)	2.74 (1.47–5.13)

HR=hazard ratio; CI=confidence interval; Ref=reference; NA=not applicable. *Adjusted for age/sex, education level, family history of lung cancer in first-degree relatives, physical activity, alcohol consumption, history of chronic respiratory diseases, occupational exposure to hazardous substances, secondhand smoke exposure, solid fuel used for heating in winter, solid fuel used for cooking, cooking oil fume exposure, and high BMI.

© 2026 Liu Y et al. *JAMA Network Open*.

eTable 14. Sensitivity analysis of association between smoking status and LC incidence risk, by follow-up duration, using a parsimonious model

	Events	Person-years	Crude HR (95%CI)	Adjusted HR* (95%CI)
**Entire follow-up period**				
Never-smoker	20	62,745.33	Ref	Ref
Smoker	56	76,266.18	2.33 (1.40-3.88)	2.52 (1.48-4.28)
**Within 1-year follow-up duration**				
Never-smoker	5	14,675.61	Ref	Ref
Smoker	8	15,741.06	1.49 (0.49-4.56)	1.76 (0.46-4.75)
**Within 2-year follow-up duration**				
Never-smoker	14	28,059.70	Ref	Ref
Smoker	26	30,618.76	1.69 (0.88-3.24)	1.85 (0.94-3.63)
**Within 3-year follow-up duration**				
Never-smoker	15	38,380.16	Ref	Ref
Smoker	35	43,533.35	2.06 (1.12-3.77)	2.21 (1.18-4.14)
**Within 4-year follow-up duration**				
Never-smoker	17	46,372.45	Ref	Ref
Smoker	43	54,699.76	2.16 (1.23-3.78)	2.34 (1.31-4.18)
**Within 5-year follow-up duration**				
Never-smoker	18	52,335.47	Ref	Ref
Smoker	49	63,084.79	2.28 (1.33-3.91)	2.46 (1.40-4.30)

HR=hazard ratio; CI=confidence interval; Ref=reference. *Adjusted for age, sex, family history of LC in first-degree relatives, history of chronic respiratory diseases.

© 2026 Liu Y et al. *JAMA Network Open*.

eTable 15. Sensitivity analysis of association between smoking status and LC incidence risk, by sex and age, using a parsimonious model

	Events	Person-years	Crude HR (95%CI)	Adjusted HR* (95%CI)
**By sex**				
**Male**				
Never-smoker	1	1,696.89	Ref	Ref
Smoker	50	67,028.04	1.28 (0.18–9.36)	1.12 (0.15–8.25)
**Female**				
Never-smoker	19	61,048.44	Ref	Ref
Smoker	6	9,238.13	2.27 (0.90–5.72)	2.18 (0.86–5.56)
**By age, year**				
**40–49**				
Never-smoker	1	13,409.76	Ref	Ref
Smoker	4	18,053.14	2.88 (0.32–25.73)	2.50 (0.26–24.42)
**50–54**				
Never-smoker	5	14,251.89	Ref	Ref
Smoker	9	16,157.13	1.58 (0.46–5.41)	1.66 (0.55–5.02)
**55–74**				
Never-smoker	14	35,083.68	Ref	Ref
Smoker	43	42,055.91	2.57 (1.41–4.71)	2.74 (1.49–5.05)

HR=hazard ratio; CI=confidence interval; Ref=reference. *Adjusted for age/sex, family history of LC in first-degree relatives, history of chronic respiratory diseases.

© 2026 Liu Y et al. *JAMA Network Open*.

eTable 16. Sensitivity analysis of association between smoking pack-years and LC incidence risk, by follow-up duration, using a parsimonious model

	Events	Person-years	Crude HR (95%CI)	Adjusted HR* (95%CI)
**Entire follow-up period**				
0 pack-years (Never-smoker)	20	62,745.33	Ref	Ref
<20 pack-years	4	15,358.07	0.83 (0.28-2.44)	0.84 (0.28–2.45)
20–<30 pack-years	10	14,647.46	2.17 (1.02-4.65)	2.38 (1.10–5.15)
≥30 pack-years	42	46,061.58	2.87 (1.68-4.88)	3.03 (1.76–5.19)
**Within 1-year follow-up duration**				
0 pack-years (Never-smoker)	5	14,675.61		Ref
<20 pack-years	2	2,798.75	2.09 (0.58-7.53)	1.96 (0.56–6.86)
20–<30 pack-years	2	2,684.45	2.18 (0.67-6.99)	2.22 (0.67–7.36)
≥30 pack-years	4	10,222.92	1.15 (0.31-4.27)	1.15 (0.31–4.30)
**Within 2-year follow-up duration**				
0 pack-years (Never-smoker)	14	28,059.70		Ref
<20 pack-years	3	5,526.34	1.07 (0.31-3.74)	1.07 (0.31–3.75)
20–<30 pack-years	8	5,326.39	2.97 (0.87-10.14)	3.00 (0.89–10.12)
≥30 pack-years	15	19,698.89	1.52 (0.73-3.15)	1.59 (0.76–3.31)
**Within 3-year follow-up duration**				
0 pack-years (Never-smoker)	15	38,380.16		Ref
<20 pack-years	3	8,115.10	0.95 (0.27-3.29)	0.92 (0.27–3.20)
20–<30 pack-years	10	7,888.12	3.26 (1.46-7.26)	3.49 (1.55–7.85)
≥30 pack-years	22	27,432.63	2.05 (1.06-3.95)	2.12 (1.10–4.12)
**Within 4-year follow-up duration**				
0 pack-years (Never-smoker)	17	46,372.45		Ref
<20 pack-years	3	10,495.53	0.79 (0.23-2.69)	0.77 (0.23–2.64)
20–<30 pack-years	10	10,253.23	2.69 (1.23-5.89)	2.94 (1.33–6.50)
≥30 pack-years	30	33,824.64	2.42 (1.34-4.40)	2.55 (1.40–4.66)
**Within 5-year follow-up duration**				
0 pack-years (Never-smoker)	18	52,335.47		Ref
<20 pack-years	3	12,365.64	0.72 (0.21-2.44)	0.71 (0.21–2.42)
20–<30 pack-years	10	12,006.75	2.46 (1.13-5.33)	2.67 (1.22–5.84)
≥30 pack-years	36	38,559.92	2.72 (1.54-4.79)	2.85 (1.61–5.06)

HR=hazard ratio; CI=confidence interval; Ref=reference. *Adjusted for age, sex, family history of LC in first-degree relatives, history of chronic respiratory diseases.

© 2026 Liu Y et al. *JAMA Network Open*.

eTable 17. Sensitivity analysis of association between smoking pack-years and LC incidence risk, by sex and age, using a parsimonious model

	Events	Person-years	Crude HR (95%CI)	Adjusted HR* (95%CI)
**By sex**				
**Male**				
0 pack-years (Never-smoker)	1	1,696.89	Ref	Ref
<20 pack-years	3	10,071.73	0.51 (0.05–4.92)	0.42 (0.04–4.12)
20–<30 pack-years	9	13,397.69	1.15 (0.14–9.11)	1.01 (0.12–8.14)
≥30 pack-years	38	43,447.45	1.50 (0.20–10.97)	1.29 (0.17–9.59)
**Female**				
0 pack-years (Never-smoker)	19	61,048.44	Ref	Ref
<20 pack-years	1	5,286.34	0.67 (0.09–5.01)	0.66 (0.09–4.97)
20–<30 pack-years	1	1,249.77	2.82 (0.38–21.11)	2.83 (0.38–21.24)
≥30 pack-years	4	2,614.13	5.19 (1.76–15.30)	4.62 (1.55–13.77)
**By age, year**				
**40–49**				
0 pack-years (Never-smoker)	1	13,409.76	Ref	Ref
<20 pack-years	0	5,515.53	NA	NA
20–<30 pack-years	3	5,405.17	7.00 (0.73–67.31)	6.70 (0.62–72.77)
≥30 pack-years	1	7,078.17	1.84 (0.11–29.39)	1.70 (0.10–28.58)
**50–54**				
0 pack-years (Never-smoker)	5	14251.89	Ref	Ref
<20 pack-years	0	3410.7315	NA	NA
20–<30 pack-years	0	2914.3123	NA	NA
≥30 pack-years	9	9802.3945	2.64 (0.88–7.89)	2.67 (0.95–7.51)
**55–74**				
0 pack-years (Never-smoker)	14	35,083.68	Ref	Ref
<20 pack-years	4	6,431.80	1.56 (0.51–4.75)	1.57 (0.51–4.77)
20–<30 pack-years	7	6,327.98	2.78 (1.12–6.91)	3.06 (1.22–7.68)
≥30 pack-years	32	29,181.01	2.76 (1.47–5.17)	2.98 (1.58–5.63)

HR=hazard ratio; CI=confidence interval; Ref=reference; NA=not applicable. *Adjusted for age/sex, family history of LC in first-degree relatives, history of chronic respiratory diseases.

© 2026 Liu Y et al. *JAMA Network Open.*

eTable 18. Association between smoking cessation and lung cancer risk

	Events	Person-years	Incidence rate per 100,000 person-years	Crude HR (95%CI)	Adjusted HR* (95%CI)
**Quit smoking <5 years**	1	4,271.82	23.41	0.31 (0.04–2.25)	0.31 (0.04–2.23)
**Quit smoking 5–<15 years**	2	2,244.30	89.11	1.17 (0.28–4.80)	1.13 (0.27–4.71)
**Quit smoking ≥15 years**	0	404.63	0	NA	NA
**Current smoking**	53	69,345.43	76.43	Ref	Ref

HR=hazard ratio; CI=confidence interval; Ref=reference; NA=not applicable.*Adjusted for pack-years, age, sex, education level, family history of lung cancer in first-degree relatives, physical activity, alcohol consumption, history of chronic respiratory diseases, occupational exposure to hazardous substances, solid fuel used for heating in winter, solid fuel used for cooking, cooking oil fume exposure, and high BMI.

eTable 19. Sensitivity analysis of association between smoking cessation and lung cancer risk among smokers, after excluding participants with <3 years of follow-up

	Events	Person-years	Incidence rate per 100000 person-years	Crude HR (95%CI)	Adjusted HR* (95%CI)
**Quit smoking <5 years**	1	3,850.90	25.97	0.32 (0.04-2.29)	0.32 (0.04-2.29)
**Quit smoking 5–<15 years**	2	1,682.40	118.88	1.45 (0.35-5.94)	1.45 (0.34-6.10)
**Quit smoking ≥15 years**	0	211.14	0.00	NA	NA
**Current smoking**	53	63,197.78	83.86	Ref	Ref

HR=hazard ratio; CI=confidence interval; Ref=reference; NA=not applicable. *Adjusted for age, sex, education level, family history of lung cancer in first–degree relatives, physical activity, alcohol consumption, history of chronic respiratory diseases, occupational exposure to hazardous substances, solid fuel used for heating in winter, solid fuel used for cooking, cooking oil fume exposure, and high BMI.

eTable 20. Sensitivity analysis of association between smoking cessation and lung cancer risk among smokers, after accounting for competing risks

	Events	Person-years	Incidence rate per 100000 person-years	Crude HR (95%CI)	Adjusted HR* (95%CI)
**Quit smoking <5 years**	1	4,271.82	23.41	0.31 (0.04–2.23)	0.31 (0.04–2.25)
**Quit smoking 5–<15 years**	2	2,244.30	89.11	1.17 (0.28–4.81)	1.13 (0.26–4.95)
**Quit smoking ≥15 years**	0	404.63	0	NA	NA
**Current smoking**	53	69,345.43	76.43	Ref	Ref

HR=hazard ratio; CI=confidence interval; Ref=reference; NA=not applicable. *Adjusted for age, sex, education level, family history of lung cancer in first–degree relatives, physical activity, alcohol consumption, history of chronic respiratory diseases, occupational exposure to hazardous substances, solid fuel used for heating in winter, solid fuel used for cooking, cooking oil

© 2026 Liu Y et al. *JAMA Network Open*.

fume exposure, and high BMI.

eTable 21. Sensitivity analysis of association between smoking cessation and LC incidence risk, using a parsimonious model

	Events	Person-years	Crude HR (95%CI)	Adjusted HR* (95%CI)
**Quit smoking <5 years**	1	4,271.82	0.31 (0.04–2.25)	0.31 (0.04–2.27)
**Quit smoking 5–<15 years**	2	2,244.30	1.17 (0.28–4.80)	1.11 (0.27–4.58)
**Quit smoking ≥15 years**	0	404.63	NA	NA
**Current smoking**	53	69,345.43	Ref	Ref

HR=hazard ratio; CI=confidence interval; Ref=reference; NA=not applicable.*Adjusted for age, sex, family history of LC in first-degree relatives, history of chronic respiratory diseases.

© 2026 Liu Y et al. *JAMA Network Open*.
