# Supplementary material for: Tobacco Smoking and Lung Cancer Risk After Negative Baseline Low-Dose Computed Tomography Findings
Source: JAMA Netw Open. 2026 Mar 20;9(3):e261913. doi: 10.1001/jamanetworkopen.2026.1913 (PMC13005165; doi:10.1001/jamanetworkopen.2026.1913)
Supplement: Supplement 2. — Data Sharing Statement [file jamanetwopen-e261913-s002.pdf]

Data Sharing Statement

Liu. Tobacco Smoking and Lung Cancer Risk After Negative Baseline Low-Dose Computed Tomography Findings. *JAMA Netw Open*. Published March 20, 2026.
doi:10.1001/jamanetworkopen.2026.1913

Data
Data available: Yes
Data types: Participant data with identifiers
How to access data: The datasets used and/or analyzed during the current study are available from the corresponding author （shaokaizhang@12.com) on reasonable request.
When available: With publication

Supporting Documents
Document types: Statistical/analytic code
How to access documents: The codes used during the current study are available from the corresponding author （shaokaizhang@12.com) on reasonable request.
When available: With publication

Additional Information
Who can access the data: Researchers whose proposed use of the data has been approved
Types of analyses: for any purpose or for a specified purpose
Mechanisms of data availability: with a signed data access agreement
